# Primaquine-Induced Methemoglobinemia: A Case Report

**DOI:** 10.7759/cureus.99414

**Published:** 2025-12-16

**Authors:** Rita Leite Cruz, César B Vieira, Pedro Silva, Rui Pereira, Nuno Germano

**Affiliations:** 1 Intensive Care Service, São José Local Health Unit, Lisbon, PRT

**Keywords:** critical care, hypoxia, immunosuppression, methemoglobinemia, pneumocystis jirovecii pneumonia

## Abstract

Methemoglobinemia is a life-threatening side effect of several drugs, including primaquine. When endogenous counter-oxidative stress mechanisms are overwhelmed, hemoglobin is oxidized to methemoglobin. This "oxygen scavenger" leads to tissue hypoxia, despite adequate alveolar gas exchange.

A 47-year-old male under immunosuppression after a reno-pancreatic transplant was admitted to the ICU for respiratory failure following suspected *Pneumocystis jirovecii* pneumonia (PJP), and empiric treatment was started. After ICU admission, treatment with cotrimoxazole was switched to primaquine due to hematological toxicity. Progressively, the oxygenated hemoglobin fraction declined despite normal PaO_2_ levels. Concurrently, methemoglobin levels rose, suggesting primaquine as the culprit. Treatment was switched to pentamidine, and ascorbic acid was administered. Methemoglobin levels subsequently lowered, and oxygen saturation normalized. G6PDH activity levels were within the normal range. Pentamidine was continued for a total of 21 days while the patient slowly recovered.

Methemoglobinemia is a rare complication of primaquine treatment with severe consequences. A discrepancy between PaO_2_ and the oxygenated hemoglobin fraction should raise awareness of this diagnosis and prompt immediate action.

## Introduction

*Pneumocystis jirovecii* pneumonia (PJP) is a fungal infection caused by *Pneumocystis jirovecii*, primarily affecting immunocompromised individuals such as those with HIV infection, malignancy, or organ transplants. Its symptoms are similar to other pneumonias and typically include fever, cough, dyspnea, and hypoxia [1,2]. Radiologically, PJP classically presents with diffuse bilateral interstitial or ground-glass infiltrates on chest imaging [1,3]. Diagnosis is established through a combination of clinical suspicion, radiologic findings, and laboratory confirmation by PCR or microscopy of respiratory specimens [1,3]. Treatment relies on high-dose trimethoprim-sulfamethoxazole as first-line therapy, with pentamidine or atovaquone as alternatives in case of intolerance or resistance [1-3]. The definition of refractory PJP is based on the absence of clinical improvement or worsening of respiratory function, documented by arterial blood gases, after four to eight days of specific therapy, usually trimethoprim-sulfamethoxazole, provided that concomitant infectious or noninfectious causes have been excluded. According to the Infectious Diseases Society of America and the Office of AIDS Research Advisory Council guidelines, clinicians should await this period before considering a change in therapy for treatment failure, as early and reversible deterioration within the first three to five days may occur due to the inflammatory response associated with organism lysis [4]. Adjunctive corticosteroids are recommended in moderate-to-severe cases with arterial oxygen pressure below 70 mmHg to reduce inflammation and improve outcomes [1].

Methemoglobinemia is a disorder characterized by elevated levels of methemoglobin, a form of hemoglobin in which the ferrous iron (Fe²⁺) in the heme group is oxidized to ferric iron (Fe³⁺), rendering that fraction unable to bind oxygen and reducing the overall oxygen-carrying capacity of the blood [5,6]. Consequently, tissue hypoxia develops despite normal or even elevated arterial oxygen tension (PaO₂), not only because methemoglobin cannot carry oxygen but also because the remaining ferrous hemoglobin exhibits a leftward shift of the oxyhemoglobin dissociation curve, impairing oxygen release to peripheral tissues [6]. Total hemoglobin consists of several fractions (oxyhemoglobin, deoxyhemoglobin, carboxyhemoglobin, and methemoglobin), quantified during arterial blood gas analysis [5,7]. However, conventional pulse oximetry, which uses two wavelengths (approximately 660 nm and 940 nm) to detect oxyhemoglobin and deoxyhemoglobin, cannot differentiate methemoglobin or carboxyhemoglobin. Because methemoglobin absorbs light at both wavelengths nearly equally, the pulse oximeter tends to display falsely stable readings around 82-86%, regardless of the true oxygen saturation. This results in a characteristic discrepancy, known as the "saturation gap," between the pulse oximetry reading (SpO₂) and the arterial oxygen saturation measured by blood gas analysis, which serves as a key diagnostic clue [6,7]. Clinically, methemoglobinemia manifests with cyanosis unresponsive to oxygen, chocolate-brown blood, metabolic acidosis, and tachycardia [5,6]. Symptom severity correlates with methemoglobin levels: mild dyspnea, nausea, and tachycardia typically appear above 30%, whereas neurologic depression, lethargy, or stupor occur when levels exceed 50% [6,8].

Methemoglobinemia develops when endogenous antioxidant systems are overwhelmed and hemoglobin oxidation exceeds reduction capacity [5]. The condition shifts the oxyhemoglobin dissociation curve to the left, reducing oxygen release to tissues and worsening hypoxia [6,8]. It may be congenital, due to cytochrome b₅ reductase deficiency or hemoglobin M variants, or acquired, typically resulting from exposure to oxidizing agents such as dapsone, nitrates, benzocaine, or primaquine [5,7]. Management includes discontinuation of the offending agent and administration of methylene blue, which acts as an artificial electron carrier to restore hemoglobin to its reduced state. In mild cases, or when methylene blue is ineffective or contraindicated, such as in patients with glucose-6-phosphate dehydrogenase (G6PD) deficiency, ascorbic acid may be used as an alternative or adjunctive reducing agent, as it promotes the slow nonenzymatic reduction of methemoglobin independently of NADPH (nicotinamide adenine dinucleotide phosphate) availability [6,8-10].

Glucose-6-phosphate dehydrogenase (G6PD) deficiency is an important predisposing factor for both hemolytic anemia and methemoglobinemia. G6PD catalyzes the first step of the pentose phosphate pathway, generating NADPH, which maintains glutathione in its reduced form and enables cytochrome b₅ reductase to convert methemoglobin back to hemoglobin [9,10]. In G6PD-deficient individuals, the lack of sufficient NADPH compromises this reduction process, leading to the accumulation of methemoglobin and consequent tissue hypoxia [9]. Drug-induced oxidative stress, caused by sulfonamides, dapsone, or fava beans, can precipitate hemolysis or methemoglobinemia in such patients [10,11].

During an acute hemolytic crisis, G6PD activity may appear falsely normal due to reticulocytosis, since younger erythrocytes contain higher enzyme levels [10]. Therefore, testing should be repeated several weeks after recovery to confirm the diagnosis [10,11]. Awareness of G6PD deficiency is clinically essential, as methylene blue administration in these patients can paradoxically worsen hemolysis and methemoglobinemia due to impaired NADPH generation [9-11].

This article was previously presented as a meeting abstract at the 2024 IX Jornadas Técnicas de Medicina Intensiva.

## Case presentation

A 46-year-old man with a relevant medical history of combined kidney-pancreas transplantation one year prior to this admission, under immunosuppressive therapy with tacrolimus, mycophenolic acid, prednisolone, and thymoglobulin, with drug levels within therapeutic ranges and no recent adjustments; type 1 diabetes mellitus diagnosed at age eight with target-organ damage, namely diabetic nephropathy requiring dialysis at age 30, proliferative diabetic retinopathy with previous surgical intervention, and diabetic polyneuropathy with transient paresthesias in the lower limbs; arterial hypertension treated since age 21, dyslipidemia, and cytomegalovirus (CMV) positivity two months before admission, on prophylactic-dose valganciclovir at the time.

**Figure 1:**
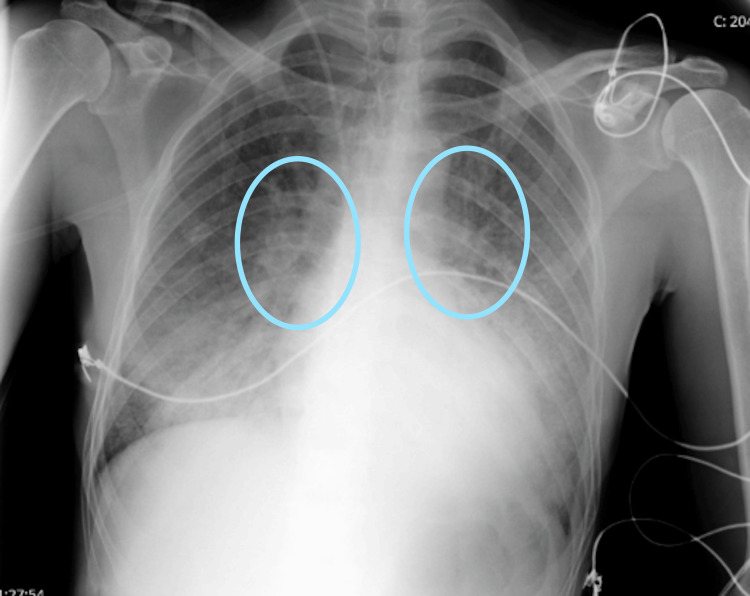
Chest X-ray on admission showing bilateral perihilar interstitial opacities, marked with blue ovals, consistent with diffuse pulmonary involvement in an immunocompromised patient.

At the elective kidney transplant follow-up consultation, he presented with a two-week history of fever and fatigue, progressively worsening with exertional dyspnea for one week and non-productive cough. No other associated symptoms were reported. On presentation, laboratory findings showed leukopenia (3,000/µL; 4,000-11,000/µL) with 37% neutrophils (40-60%), CRP (C-reactive protein) 24 mg/dL (<0.5 mg/dL), worsening renal function with creatinine 2.5 mg/dL (0.6-1.2 mg/dL; baseline 1 mg/dL), and LDH (lactate dehydrogenase) 455 U/L (140-280 U/L), as shown in Table 1. Arterial blood gas analysis was compatible with type I respiratory failure, and chest X-ray revealed bilateral infiltrates without consolidation (Figure 1). Pneumonia in an immunocompromised patient was diagnosed, with suspicion of Pneumocystis jirovecii infection, along with other possibilities such as atypical bacterial pneumonia or CMV pneumonitis. Empiric therapy was initiated with levofloxacin, cotrimoxazole, and ganciclovir.

**Table 1:** Admission laboratory results, including reference ranges.

Parameter	Result	Reference range
Hemoglobin	14.8 g/dL	13-17 g/dL
Platelets	281 x 10^9^/L	150-450 x 10^9^/L
Leukocytes	3,000/µL	4,000–11,000/µL
Neutrophils	37%	40–60%
C-reactive protein	24 mg/dL	<0.5 mg/dL
Creatinine	2.5 mg/dL	0.6–1.2 mg/dL
Urea	156 mg/dL	19.1-44.1 mg/dL
Lactate dehydrogenase	455 U/L	140–280 U/L

**Figure 2:**
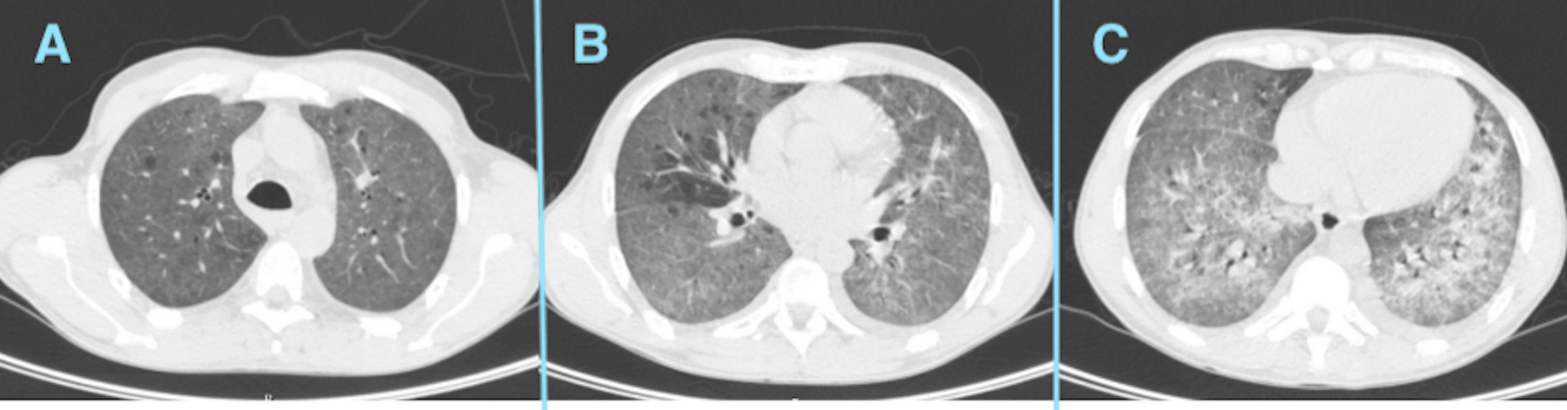
Bilateral ground-glass opacities on chest computed tomography at intensive care unit admission. (A) Axial CT image showing patchy bilateral ground-glass opacities predominantly in the upper lung fields, with preserved bronchovascular markings. (B) Progression of bilateral ground-glass opacities with increasing confluence and extension toward the perihilar regions. (C) More advanced bilateral ground-glass opacification with superimposed interlobular septal thickening, resulting in a “crazy-paving” appearance.

Bronchofibroscopy was performed upon admission, with subsequent isolation of *Pneumocystis jirovecii*. No images are available, but the examination showed pale and friable mucosa with diffuse inflammatory changes and patches of whitish, foamy material adhering to the bronchial walls, findings consistent with *P. jirovecii* infection. On the third hospital day, the patient clinically deteriorated, requiring escalation of oxygen therapy and transfer to the intensive care unit (ICU) for noninvasive ventilation. At that time, chest CT (Figure 2) showed reduced parenchymal aeration with bilateral "ground-glass" opacities.

Upon ICU admission, repeat bronchoscopy revealed diffuse mucosal edema, and noninvasive ventilation was initiated (support 8, EPAP 6, FiO₂ 75%). Progressive clinical deterioration led to orotracheal intubation on the fifth hospital day due to respiratory exhaustion. Sedation was adjusted accordingly, and invasive mechanical ventilation was maintained with FiO₂ 80-100%. Given the absence of clinical improvement and inability to reduce ventilatory support, therapeutic failure was assumed on the eighth day of cotrimoxazole treatment, which was then replaced with clindamycin and primaquine. Neuromuscular blockade and prone positioning were initiated, but there was no significant improvement. The course was complicated by *Escherichia coli* bacteremia, prompting targeted antibiotic therapy.

**Figure 3:**
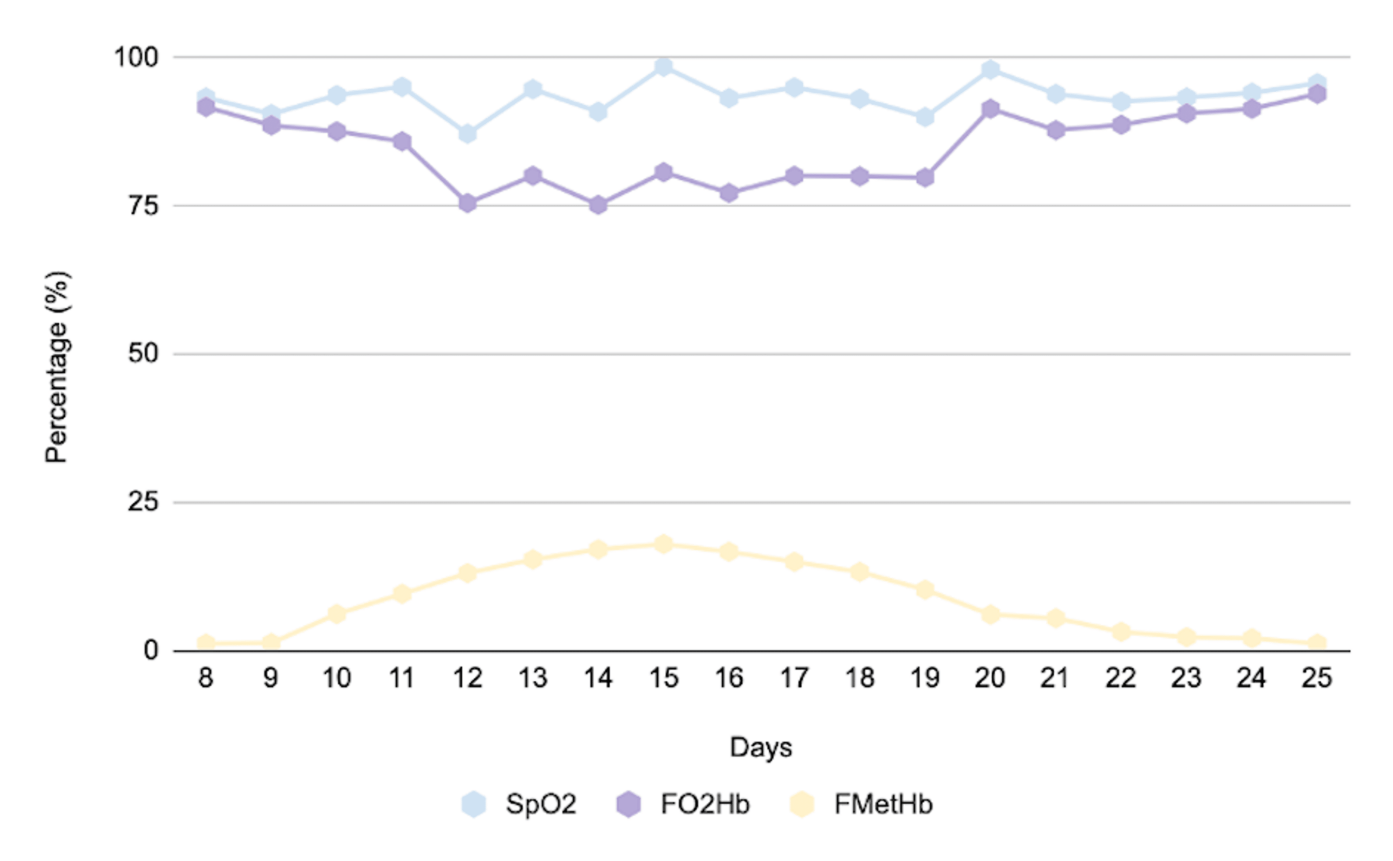
Graph showing the progression of methemoglobin (MetHb), peripheral oxygen saturation (SpO₂), and oxyhemoglobin fraction (FO₂Hb) levels.

On the 14th day of hospitalization, the arterial oxygen partial pressure values no longer correlated with pulse oximetry, showing decreasing oxyhemoglobin levels and a progressive increase in methemoglobin (Figure 3).

**Figure 4:**
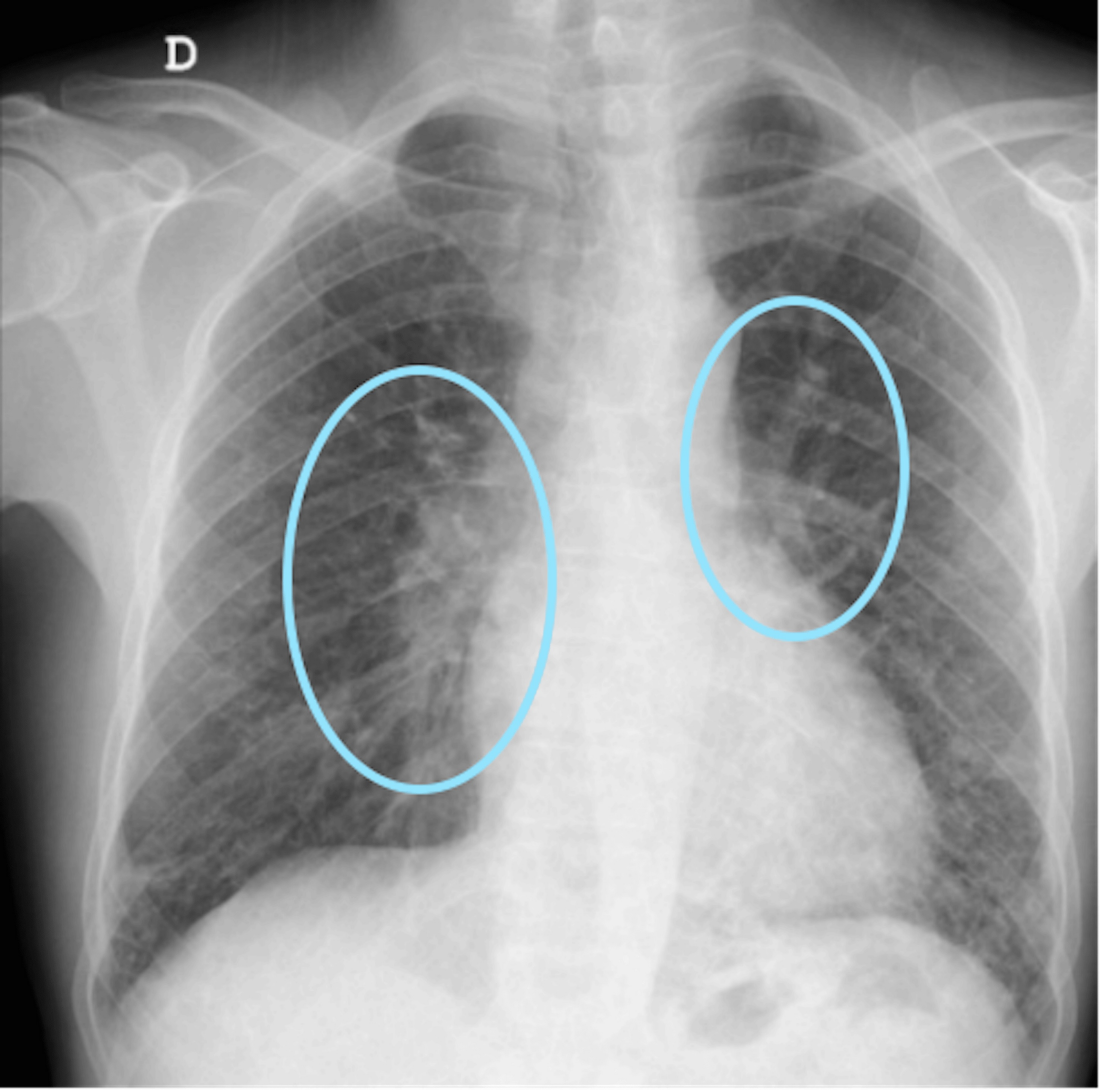
Follow-up chest X-ray one year after the acute episode, showing complete radiographic resolution of the previously observed bilateral perihilar opacities (highlighted areas).

The diagnosis of methemoglobinemia was confirmed by measuring the methemoglobin fraction in an arterial blood gas analysis. Other differential diagnoses, such as carbon monoxide poisoning or sulfhemoglobinemia, were considered due to the dissociation between oxygen saturation and the oxyhemoglobin fraction. Among possible etiologies, primaquine was deemed the most likely cause and was replaced by pentamidine. G6PDH activity was measured, and ascorbic acid (1 g every six hours) was started while awaiting results. With clinical improvement and normalization of oxygen saturation and methemoglobin levels, no further intervention was required. Pentamidine therapy was continued for a total of 21 days, and the patient achieved full recovery with positive radiographic control (Figure 4).

## Discussion

This case illustrates a complex presentation of pneumonia in an immunocompromised, HIV-negative patient, a setting that requires a broad differential diagnosis and early empiric broad-spectrum antimicrobial therapy, often associated with adverse effects. After microbiological confirmation, treatment was directed toward PJP, a condition with significantly higher mortality in non-HIV immunocompromised hosts [12]. Clinical deterioration despite first-line trimethoprim-sulfamethoxazole, known to markedly worsen prognosis, led to timely escalation to a second-line regimen with clindamycin and primaquine, followed by the development of methemoglobinemia, a recognized adverse reaction to primaquine.

Severe methemoglobinemia, defined as metHb levels above 30%, or between 20% and 30% with hypoxia, is a medical emergency with potentially life-threatening consequences [6-8]. Management requires immediate discontinuation of the offending drug. In this case, primaquine was replaced by pentamidine, and ascorbic acid was administered instead of methylene blue due to unknown G6PD status, avoiding the risk of hemolysis. Subsequent testing showed normal G6PD activity, confirming that methylene blue could have been safely used.

The dissociation between pulse oximetry and arterial blood gas analysis was explained by the optical limitations of standard two-wavelength oximeters, which cannot distinguish methemoglobin from oxyhemoglobin and deoxyhemoglobin. Because methemoglobin absorbs light almost equally at both wavelengths, SpO₂ readings remain falsely stable around 85%, producing the characteristic "saturation gap" despite reduced true oxyhemoglobin (FO₂Hb).

In HIV-negative immunocompromised patients, PJP mortality ranges from 30% to 50%, compared with 10% to 20% in HIV-positive individuals [12]. The favorable outcome observed here reflects the early diagnosis of PJP, timely recognition of treatment failure, and appropriate management of the ensuing complication. Combination therapy with primaquine and clindamycin remains the preferred alternative for refractory PJP, though its hematologic toxicity, particularly hemolysis and methemoglobinemia, requires vigilant monitoring [13]. Similar cases of primaquine-induced methemoglobinemia have been reported, typically presenting with unexplained hypoxia and a saturation gap, resolving after discontinuation of the drug [14]. Our findings align with this evidence and underscore the diagnostic challenge of identifying methemoglobinemia in critically ill patients.

Finally, screening for G6PD deficiency before initiating oxidant drugs such as primaquine or dapsone is recommended by both the CDC and WHO, though it is frequently omitted in urgent clinical settings, exposing patients to preventable complications [15].

## Conclusions

Methemoglobinemia is a rare complication of primaquine treatment. The severity of this condition depends on the time between the onset of symptoms and the initiation of treatment. A discrepancy between FO_2_Hb and oxygen saturation should raise suspicion for this diagnosis. The precipitating drug should be discontinued, and specific treatment is warranted for methemoglobin levels above 20%. It is advisable to obtain G6PD activity levels before starting treatment with high-risk drugs, such as primaquine.
